# Outcomes of Atrioventricular Node Ablation and Pacing in Patients with Heart Failure and Atrial Fibrillation: From Cardiac Resynchronization Therapy to His Bundle Pacing

**DOI:** 10.3390/jcdd10070272

**Published:** 2023-06-26

**Authors:** Ioanna Koniari, Andreas Gerakaris, Nicholas Kounis, Dimitrios Velissaris, Archana Rao, Mark Ainslie, Ahmed Adlan, Panagiotis Plotas, Ignatios Ikonomidis, Virginia Mplani, Ming-Yow Hung, Cesare de Gregorio, Theofilos Kolettis, Dhiraj Gupta

**Affiliations:** 1 Department of Electrophysiology, Liverpool Heart and Chest Hospital, Liverpool L14 3PE, UK; iokoniari@yahoo.gr (I.K.); archana.rao@lhch.nhs.uk (A.R.); dhiraj.gupta@lhch.nhs.uk (D.G.); 2 Department of Internal Medicine, University Hospital of Patras, 26500 Patras, Greece; agerakaris72@gmail.com (A.G.); dimitrisvelissaris@yahoo.com (D.V.); 3 Department of Medicine, Division of Cardiology, University Hospital of Patras, 26500 Patras, Greece; 4 Department of Cardiology, Manchester Heart Institute, University Hospital of Manchester, Manchester M23 9LT, UK; mark.ainslie@mft.nhs.uk (M.A.); adlan.ahmed@gmail.com (A.A.); 5 Laboratory Primary Health Care, School of Health Rehabilitation Sciences, University of Patras, 26500 Patras, Greece; pplotas@upatras.gr; 6 2nd Department of Cardiology, “Attikon” Hospital, National and Kapodistrian University of Athens Medical School, 12462 Athens, Greece; ignoik@gmail.com; 7 Department of Intensive Care Unit, Patras University Hospital, 26500 Patras, Greece; virginiamplani@yahoo.gr; 8 Division of Cardiology, Department of Internal Medicine, Shuang Ho Hospital, Taipei Medical University, No.291, Zhongzheng Rd., Zhonghe District, New Taipei City 23561, Taiwan; myhung6@ms77.hinet.net; 9 Taipei Heart Institute, Taipei Medical University, Taipei City 110301, Taiwan; 10 Division of Cardiology, Department of Internal Medicine, School of Medicine, College of Medicine, Taipei Medical University, Taipei City 110301, Taiwan; 11 Department of Clinical and Experimental Medicine, University of Messina Medical School, 98122 Messina, Italy; cesare.degregorio@unime.it; 12 Cardiovascular Research Institute, Department of Cardiology, Medical School, University of Ioannina, 45110 Ioannina, Greece; theofilos.m.kolettis@gmail.com

**Keywords:** atrioventricular node ablation, His bundle pacing, cardiac resynchronization therapy, heart failure, atrial fibrillation

## Abstract

**Objective:** To review the relevant literature on the use of atrioventricular node ablation and pacing in patients with heart failure and atrial fibrillation. **Methods:** APubMed/MEDLINE and SCOPUS search was performed in order to assess the clinical outcomes of atrioventricular node ablation and pacemaker implantation, as well as the complications that may occur. **Results:** Several clinical trials, observational analyses and meta-analyses have shown that the “pace and ablate” strategy not only improves symptoms but also can enhance cardiac performance in patients with heart failure and atrial fibrillation. Although this procedure is effective and safe, some complications may occur including worsening of heart failure, permanent fibrillation, arrhythmias and sudden death. Regarding pacemaker implantation, cardiac resynchronization therapy is shown to be the optimal choice compared to right ventricle apical pacing. His bundle pacing is a promising alternative to cardiac resynchronization therapy and has shown beneficial effects, while left bundle branch pacing is an innovative modality. **Conclusions:** Atrioventricular node ablation and pacemaker implantation is shown to have beneficial effects on clinical outcomes of patients with atrial fibrillation ± heart failure who do not respond or are intolerant to medical treatment. Cardiac resynchronization therapy is the treatment of choice and His bundle pacing seems to be an effective alternative way of pacing in these patients.

## 1. Introduction

Heart Failure (HF) and Atrial Fibrillation (AF) are two cardiovascular (CV) conditions affecting many people worldwide. In recent years both conditions have increased in prevalence, probably due to a combination of increasing life expectancy and increase in comorbidities such as hypertension, diabetes and obesity. AF is the most common arrhythmia and the American Heart Association estimated that around 46.3 million people around the globe suffered with AF in 2016 (23.2 million males, 23.1 million females) [1,2]. Researchers predict that in the following decades the number of individuals with AF is going to increase, reaching 6–16 million in the USA in 2050 and 14 million in Europe in 2060 [3,4,5]. HF is a syndrome affecting many people globally. The prevalence of AF in HF is between 13% and 27% [6,7,8,9,10]. In the Framingham Heart Study, among 1470 patients with AF or HF, 383 individuals (26%) suffered from both conditions [11]. There is a connection between these two CV conditions, as they have similar risk factors, they are often concomitant, and each can result in development of the other.

Decreased cardiac output in patients with HF results in neurohormonal activation that leads to cardiac remodeling and fibrosis formation, which plays an important role in AF initiation. Additionally, in these patients there is an increased Left Ventricular Filling Pressure (LVFP) transmitted to the left atrium that leads to elevated atrial pressure and dilation. A dilated atrium is able to maintain the arrhythmia through multiple wavelets of re-entry [12]. In patients with AF, the increased resting heart rate (HR) and the exaggerated HR response during exercise result in reduced Left Ventricle (LV) filling time, absence of atrial contraction, tachycardia-induced cardiomyopathy and thus an impaired diastolic and systolic function [13]. This relation between AF and HF explains why treating one affects the other.

## 2. Pace and Ablate Strategy in AF and HF Patients

The Atrioventricular node (AV node) is the only part of the electrical conduction system of the human heart that connects the atria and ventricles. AF is characterized by chaotic and irregular impulses in the atria ranged from 300 to 600 beats per minute (bpm). Rapid ventricular rate is prevented by the AV node, which slows the rate of impulses passing to the ventricles.

Two distinct treatment strategies exist for AF. One is rhythm control and the other rate control. Rhythm control tends to be used in younger patients with paroxysmal AF, while rate control is often the preferred method for older long standing persistent AF patients. Rate control can be achieved by using a combination of beta-blockers, digoxin and non-dihydropyridine calcium channel blockers. All of these are possible options in HFpEF, while only beta-blockers and digoxin can be used in HFrEF. An alternative rate control option to medical therapy is the “pace and ablate” approach which offers a more feasible ventricular rate control and R-R interval adjustment. Current 2020 ESC guidelines recommend AV node ablation in patients with AF who are non-responders or intolerant to pharmacological treatment and are not eligible for left atrial ablation (Class IIa, Level of Evidence B) [14]. Additionally, the 2014 guidelines of the American College of Cardiology/American Heart Association (ACC/AHA) endorse AV node ablation with ventricular pacing to control heart rate in patients with HF and AF, who do not tolerate pharmacological therapy or are non-responders (Class IIa, Level of Evidence B) [15]. In recent years, radiofrequency (RF) catheter ablation of the AV junction is the preferable method.

## 3. Clinical Outcomes of AV Node Ablation

Although AV node ablation is performed in many patients with chronic drug-refractory AF and HF, only a few randomized trials have been conducted in order to compare the clinical outcomes of this procedure. There are however several non-randomized studies comparing the patients’ clinical condition at baseline with their condition after a follow-up period (Table 1).

A meta-analysis by Wood et al. included 21 studies (two randomized trials, 19 non-randomized studies) conducted in a 9-year period (from 1989 to 1998) and 1181 patients with drug-refractory atrial tachyarrhythmia, mainly AF (97%) and evaluated the clinical outcomes of AV node ablation plus pacing [16]. Fifteen studies (13 non-randomized, two randomized) and 642 patients were included in the analysis of clinical outcomes with an average follow-up duration from 48 days to 2.3 years. This analysis assessed 19 parameters containing QOL, exercise duration, symptoms, healthcare use and cardiac function. There was a significant improvement in all parameters, apart from longitudinal fractional shortening (*p* = 0.08).

A recent meta-analysis by Chatarjee et al. reviewed the efficacy, effectiveness and safety of AV nodal ablation compared to those of pharmacological rhythm control strategies [17]. The efficacy analysis included five randomized or prospective trials (*n* = 314), while the other two included 11 studies (*n* = 810) and 47 studies (*n* = 5632), respectively. AV nodal ablation was related to improvement in QOL and symptoms, while there was no significant difference in exercise duration compared to drug therapy. In patients with impaired systolic function at baseline who underwent ablation, there was a significant increase in EF compared to those treated with medication (+4%; 95% CI 3.11–4.89). Moreover, all-cause mortality was equivalent in both AV node ablation and drug therapy (RR 1.05; 95% CI 0.29–3.85) and there were low percentages of ablation-related mortality (0.27%) and malignant arrhythmias (0.57%). At an average follow-up of 26.5 months, SCD incidence of 2.1% was reported.

Several trials have randomized patients in two groups (ablation plus pacemaker implantation group and medical therapy group) to compare the impact of these approaches on clinical outcomes. The findings of these trials were in accordance with the meta-analyses reports and showed that AV node ablation is associated with less symptoms compared to medical therapy. The results of these studies are summarized in Table 1.

AV node ablation and pacemaker implantation demonstrate several beneficial effects on clinical outcomes in these patients compared to pharmacological rate control. Data collected by using different kinds of questionnaires showed an improvement in QOL and symptoms. Symptoms were reduced, presumably due to ventricular rate adjustment and less side effects of pharmacological therapy. LVEF improvement was associated, mainly, with an improvement in diastolic filling and the cessation of negative inotropic medication, resulting in increasing of cardiac output and exercise duration.

## 4. Survival Analysis Post AV Node Ablation

In the meta-analysis by Wood et al.,1073 patients from 16 studies were included in the mortality analysis with a mean follow-up between 3 months and 2.3 years. The estimated monthly and 1-year mortality rates were 1.4% (95% CI 0.04–2.4) and 6.3% (95% CI 5.5–7.2), respectively [16]. Ozcan et al. assessed the mortality rate in 350 patients in Minnesota [24]. During a mean 36-month follow-up 78 patients died. There was not a significant difference in survival rates between the AV node ablation group and the controls (RR 1.14; 95% CI 0.81–1.60; *p* = 0.44). Additionally, they found that previous MI, history of CHF and medical treatment after ablation were independent factors of death (*p* < 0.001, *p* = 0.02, *p* = 0.03, respectively).

In another study, 359 patients with refractory AF were treated with RF ablation of AV junction plus pacing. Forty six of them died within a mean 41-month follow-up. One-year and five-year survival probability for all patients were 0.953 and 0.827, respectively. They also found that age ≥ 65 years (HR 1.92; 95% CI 1.00–3.69), presence of HF (HR 3.93; 95% CI 1.87–7.86), diabetes (HR 2.91; 95% CI 1.47–5.77) and fractional shortening ≤ 20% (HR 5.79; 95% CI 3.00–11.18) are independent predictors of death [25]. Darpo et al. observed 220 patients with paroxysmal or chronic AF, who underwent ablation of AV junction plus pacing [26]. Thirty-one deaths were recorded during an average 31-month follow-up period. The annual incidence of sudden death and sudden unexplained death was 1.9% and 1%, respectively.

The Atrioventricular Junction Ablation and Biventricular Pacing for Atrial Fibrillation and Heart Failure (APAF-CRT) trial enrolled patients with symptomatic, permanent AF, narrow QRS complex (≤110 ms) and at least one HF hospitalization during the last year prior to the time of inclusion [27]. In total, 133 patients were randomized into two groups: AV junction ablation+ CRT group and pharmacological rate control group with a median 29-month follow-up. Atrioventricular junction (AVJ) ablation plus CRT was superior to drug therapy inreducing both all-cause mortality (HR 0.26; 95% CI 0.10–0.65; *p* = 0.004) and the secondary combined endpoint of death of any cause or HF hospitalization (HR 0.40; 95% CI 0.22–0.73; *p* = 0.002). Regarding the primary endpoint of all-cause mortality, both patients with EF > 35% and +those with EF ≤ 35% were benefited by the AVJ ablation and biventricular (BiV) pacing (HR 0.27; 95% CI 0.08–0.84; *p* = 0.02 and HR 0.34; 95% CI 0.06–1.92; *p* = 0.22, respectively).

## 5. Deterioration of HF Post AV Node Ablation and RV Pacing

A “Pace and ablate” strategy was shown to improve cardiac performance and HF status in patients with drug-refractory AF. However, this approach has some drawbacks, especially when is followed by permanent RV apical pacing. AV node ablation has beneficial effects on cardiac function and LVEF, which are counterbalanced by the adverse hemodynamic effects and modification of the LV due to RV apical pacing. RV pacing promotes cardiac dyssynchrony and results in worsening of HF and also intensifies coexistent mitral regurgitation in patients with chronic AF [28,29].

In a study by Vanderheyden et al., 108 patients with refractory AF were treated with AV node ablation. In eight of them (~7.4%), hemodynamic deterioration was developed and was related to exacerbation of mitral regurgitation [30]. After 3 to 8 weeks from the procedure, three of these eight patients developed acute pulmonary edema and five of them congestive heart failure (CHF). It was found that patients with enlarged LV at the end of diastole and established mitral regurgitation at baseline were at increased risk.

Twidale et al. performed AV node ablation plus pacing in 44 patients with AF and CHF [31]. Four patients (~9%) developed worsening of heart failure within 24 h after the procedure. The authors referred to the observation of Vanderheyden et al. that mitral regurgitation was a result of ventricular pacing after the AV node ablation and recommended fluid restriction and administration of diuretics. Björkenheim et al. reported that in 117 who underwent a “Pace and ablate” approach, during the 58-month follow-up period, 23 of them (~20%) had at least one hospitalization due to HF [32]. Of those 23 hospitalizations, 13 (~57%) had known HF before the procedure.

## 6. Sudden Death and Ventricular Arrhythmias Post AV Node Ablation

Sudden death and ventricular arrhythmias usually occur early after the AV node ablation and pacing and are associated with failure of the pacing system, coexisting heart disease or the procedure itself. Ozcan et al. observed that in 334 patients, approximately 2.7% of them (*n* = 9) had sudden death after the procedure [25]. In 1.2% of these patients (*n* = 4) sudden death occurred the first 4 days after the ablation and was considered procedure-related. Three other patients (0.9%) died suddenly within 3 months after the procedure and were considered probably related to it. Independent predictors for sudden death included diabetes, NYHA ≥ 2, preexisting ventricular arrhythmia, valvulopathies and chronic obstructive pulmonary disease. In addition, in a recent meta-analysis the incidence of sudden cardiac death after AV junction ablation was 2.1% at an average follow-up of 26.5 months [17].

The presence of bradycardia has been shown to play an important role in the development of ventricular arrhythmias and especially VT. A case report by Peters et al. presented a patient with repeated VF which started right after the catheter ablation of AV junction [33]. This arrhythmia was pause- and bradycardia-dependent and was quelled by pacing at 90 bpm. The authors reported that this serious fatal arrhythmia may be bradycardia-induced and was not related to the ablation procedure. A study by Geelen et al. included 235 patients with refractory atrial arrhythmias (84% AF) treated with ablation [34]. A total of 100 patients had a pacing rate of ≤70 bpm and in six of them (6%) VF or sudden death was reported. In the other group of 135 patients, the pacing rate was about 90 bpm for 1–3 months after the procedure and neither VF nor sudden death occurred. Thus, they suggested that these complications can be prevented by the programming of a higher base rate, with faster rates right after the ablation. Additionally, in two other studies no early sudden death or ventricular arrhythmias were reported in patients with AF who had a pacing rate at >70 bpm for 1–3 weeks after the AV node ablation [26,35]. In a recent study by Wang et al., sudden death rate was decreased to 0.2% when pacing was set at a lower rate of 90 bpm [36].

In an ECG, ventricular repolarization is depicted by QT interval and changes in this parameter can trigger malignant arrhythmias. Cellarier et al. compared a group of 11 patients who underwent AV node ablation and received a VVI pacemaker with a control group consisted of six patients with chronic complete heart block and a VVI pacemaker [37]. This study showed an abnormal long QT interval during the first 2 days after the ablation at rates < 75 bpm, which was eliminated the following days. The authors suggested that pacing in these patients should be at ≥75 bpm, so to avoid bradycardia-dependent prolongation of QT interval and subsequent arrhythmias and sudden death [34]. Raj et al. evaluated QT dispersion during an abrupt rate drop from 80 to 40 bpm in 20 patients after ablation of AV junction [38]. Thirteen of these patients had normal LV function and seven of them had impaired LV function. A significant increase in QT dispersion was recorded in patients with LV dysfunction compared to those with normal LV function (*p* = 0.01).

Another key point in pathogenesis of ventricular arrhythmias developed after AV junction ablation is sympathetic nervous system stimulation. Hamdan et al. evaluated the sympathetic nervous system activation in ten patients with chronic AF after a successful AV ablation [39]. They reported an increase in activation of SNS in patients with pacing at 60 bpm, while at 90 bpm SNS activity was reduced.

## 7. Risk of Permanent AF Progression Post AV Node Ablation

Ablation of AV junction and permanent pacing in patients with refractory AF have been linked to more relapses of paroxysmal AF and increased incidence of permanent AF development. A study by Queiroga et al. involved 114 patients with paroxysmal AF (83%, *n* = 95) and paroxysmal AF/flutter (17%, *n* = 19) who underwent AV node ablation [40]. During a 72-month period, 52% of patients developed permanent AF, while 48% remained in sinus rhythm without a difference between AF and flutter. From the parameters evaluated at baseline, only age over 80 years seemed to be associated with progression to permanent AF but without significance (*p* = 0.07). According to Marshall et al., although AV node ablation and pacing had beneficial effects on symptoms compared to drug therapy, “ablate and pace” strategy was associated with development of persistent AF at 6 weeks (12/37 patients, 32% in ablate and pace group vs. 0/19 in drug group; *p* < 0.01) [21]. A study by Brignole et al. reported similar results to those of Marshall et al. with five out of 21 patients (24%) progressing to permanent AF 6 months after the ablation, compared to none of the patients received drug therapy [20].

The probability of progression to permanent AF after AV node ablation has been shown to rise as time goes on. Gianfranchi et al. studied 63 patients with refractory paroxysmal AF [41]. These patients were treated with AV junction ablation and dual-chamber pacemaker implantation and 22 of them (35%) developed permanent AF during an average 23-month follow-up. The authors estimated that the actual rate of permanent AF was 22%, 40% and 56% at 1-, 2- and 3-years following ablation. In a study by Gribbin et al., 42% of patients (*n* = 26/62) with paroxysmal AF, who underwent AV junction ablation developed permanent AF during a 30-month follow-up period [42]. In total, 75% of patients had developed permanent AF 86 months (~7 years) after the procedure.

Permanent pacing may have deleterious effects on cardiac function and may lead to an increased incidence of AF. RV pacing seems to be related to an increased risk of AF relapses and progression to permanent AF. A post hoc analysis of the Mode Selection Trial (MOST) assessed patients with DDDR or VVIR pacemaker and QRS duration < 120 ms at baseline [43]. In this analysis, the cumulative percent of ventricular paced (Cum%VP) beats was shown to be a predictive factor of HF hospitalizations in both DDDR and VVIR groups (HR 2.99; 95% CI 1.15–7.75 for Cum%VP > 40% and HR 2.56; 95% CI 1.48–4.43 for Cum%VP > 80%, respectively),as well as the risk of developing AF.

Apart from the effects of pacing, other factors may play an important role in developing permanent AF. Brignole et al. randomized 137 patients with symptomatic paroxysmal AF, who treated with AV node ablation and pacing, to either continuous antiarrhythmic drug therapy (*n* = 68) or not (*n* = 69) [44]. Although in the drug group there were less patients who developed permanent AF (OR 0.43; 95% CI 0.18–0.98), there was no difference in the outcomes between those progressing to chronic AF and those not. Despite the positive impact of drug therapy on developing permanent AF, patients in the drug group had more episodes of HF and hospitalizations.

## 8. Biventricular Pacing in HF and AF Post AV Node Ablation

The Dual chamber and VVI Implantable Defibrillator (DAVID) trial included 506 patients with LVEF ≤ 40% who had some indication for ICD implantation [45]. Patients were randomized to either DDDR (70 bpm) or VVI (40 bpm) pacing mode. The rates of CHF hospitalization were higher in the DDDR group (13.3% VVI vs. 22.6% DDDR; relative hazard 1.54; 95% CI 0.97–2.46). This difference was due to loss of ventricular synchrony triggered by RV apical pacing (~3% in VVI, ~56% in DDDR). A post hoc analysis of the MOST trial demonstrated the deleterious effects of RV pacing on LV function caused by electromechanical dyssynchrony between the two chambers [43]. Thus, RV pacing results in the development or worsening of HF.

Several studies have demonstrated the superiority of LV or biventricular (BiV) pacing to RV pacing in patients who underwent AV node ablation for AF (Table 2).

A study by Puggioni et al. included 44 patients with AF who underwent AV node ablation [46]. They compared RV to LV pacing performed during the first 24 h after ablation. LV pacing was associated with a more important increase in EF (17.6% in LV vs. 11.2% in RV) and a greater decrease in mitral regurgitation (16.7% in LV vs. 0% in RV). Simantirakis et al. included 12 patients (six with impaired and six with normal LV function) with permanent AF treated with AV node ablation plus pacing in their study [47]. LV-based pacing (LV free wall and BiV pacing) benefited LV contractility and LV filling in these patients compared to RV apical pacing, while there was no difference between LV free wall and BiVpacing. Leon et al. studied 20 patients with concomitant CHF and AF who initially underwent AV node ablation and RV pacing and subsequently they received a BiV pacemaker [48]. Changing to BiV pacing showed a significant increase in EF (30.9 ± 11.5% compared to 21.5 ± 6.9% at baseline; *p* < 0.001) and an improvement in NYHA functional classification (2.4 ± 0.6% compared to 3.4 ± 0.5 at baseline; *p* < 0.001).

The Post-AV Nodal Ablation Evaluation (PAVE) trial randomized 184 patients with AF,who were treated with AV node ablation, to take either aBiV (*n* = 103) or RV (*n* = 81) pacemaker [49]. At baseline, patients had a mean LVEF of 46% and 83% of them had known HF with NYHA Class II or III. At 6 months after ablation, there was a significant increase in 6-min walk distance in BiV group compared to this in RV group. Additionally, in the BiV group there was no difference in LVEF between 6 months post-ablation and baseline (46 ± 13% vs. 46 ± 16%, respectively). However, at 6 months post-ablation the EF in patients with BiV was significantly higher than the RV group (46 ± 13% vs. 41 ± 13%, respectively; *p* = 0.03). The BiV pacemaker seemed to benefit patients with EF ≤ 45% or NYHA II/III more than those with normal EF or NYHA I regarding the 6-min walk test.

A recent meta-analysis by Stavrakis et al. evaluated the impact of CRT and RV pacing on patients with AF who underwent AV node ablation [50]. This study contained five trials with 686 patients (413 patients in CRT group and 273 patients in RV group). CRT was associated with a significant decrease in HF hospitalizations (RR 0.38; 95% CI 0.17–0.85; *p* = 0.02) compared to RV pacing and with a significant change in LVEF (mean change 1.97%; 95% CI 1.52–2.42; *p* < 0.00001).

### 8.1. Indications of BiVPacing or CRT

Current 2021 ESC guidelines on heart failure recommend the use of CRT rather than RV pacing for patients with HFrEF (EF < 40%) irrespective of NYHA class or QRS width,who are candidates for ventricular pacing for high degree AV block or AF (Class I, Level of Evidence A) [51]. Additionally, according to the current 2021 ESC guidelines on cardiac pacing and cardiac resynchronizationtherapy, CRT should be considered for patients with LVEF ≤ 35% who remain in NYHA class III/IV despite the optimal medical treatment and have AF with inherent QRS ≥ 130 ms (Class IIa, Level of Evidence C) [52]. In patients with symptomatic AF and uncontrolled heart rate, who are eligible for AVJ ablation, CRT is recommended in patients with HFrEF (Class I, Level of Evidence B) and it should be preferred over RV pacing in patients with HFmrEF (Class IIa, Level of Evidence C). These recommendations differ in patients with HFpEF where there is a Class IIa, Level of Evidence B recommendation for RV pacing and a Class IIb, Level of Evidence C for CRT (Table 3).

### 8.2. His Pacing When Biventricular Pacing Fails

Although BiV pacing is superior to RV apical pacing in patients treated with AV node ablation, approximately 30% of them are non-responders. Additionally, sometimes the implantation of the LV lead (via the coronary sinus) is not possible or unsuccessful.

His-bundle pacing (HBP) offers a physiological approach to ventricular stimulation by using the native pathway of normal conduction, leading to synchronized ventricular contraction in both ventricles. In cases of failed BiV implantation or non-responders to it, HBP was shown to be efficient as an alternative option [53]. Deshmukh et al. performed permanent HBP for the first time [54]. HBP was successful in ~60% of patients with AF, dilated myocardiopathy, LVEF < 40% and NYHA III/IV and followed by ablation of AV junction. This procedure was associated with better ventricular systolic function.

Huang et al. studied 52 patients with symptomatic AF and concomitant HF who underwent AV node ablation and HBP [55]. After a 20-month follow-up a significant increase in LVEF was reported (*p* < 0.001). The reduction in HF was more notable in patients with HFrEF (*n* = 20) than in those with HFpEF (*n* = 22). HBP resulted in improvement in NYHA classification in both HFrEF (from 2.9 ± 0.6 at baseline to 1.4 ± 0.4) and HFpEF (from 2.7 ± 0.6 at baseline to 1.4 ± 0.5). In addition, the use of diuretics for management of HF was decreased in both HFrEF and HFpEF in patients with HBP.

Patients with AF and HF who underwent AV node ablation and HBP were enrolled in a recent observational study [56]. LVEF improved from 44.9 ± 14.9%at baseline to 57.6 ± 12.5% during a median follow-up of 3 years (*p* < 0.001). In addition, LVEF ≤ 40%, serum creatinine ≥ 97 μmol/L and pulmonary artery systolic pressure (PASP) ≥ 40 mmHg at baseline were associated with increased heart failure hospitalization and mortality (*p* < 0.05).

Although non-randomized observational studies have shown the feasibility of HBP plus AVNA in these patients, more data are required. Huang et al. have recently published the results of a multicenter, randomized trial in which a comparison between BiV pacing and HBP after AVNA in patients with persistent AF and reduced LVEF was performed [57]. All patients enrolled received both BiV and HBP after AVNA and then they were randomized into two groups in the phase 1 (BiV pacing or HBP the first 9 months). In the next phase patients were switched to the other pacing modality. There was a significant improvement regarding the primary endpoint (change in LVEF) in the HBP group compared with BiV pacing group (phase 1: ΔLVEF_HBP_ 21.3% and ΔLVEF_BiV pacing_ 16.7%; phase 2: ΔLVEF_HBP_3.5% and ΔLVEF_BiV pacing_−2.4%; *p* = 0.015). A significant improvement in LV end-diastolic diameter, NYHA functional class and B-type natriuretic peptide level was noted in both groups compared to baseline. However, there was no significant difference in these secondary endpoints between BiV pacing and HBP group.

**Figure 1:**
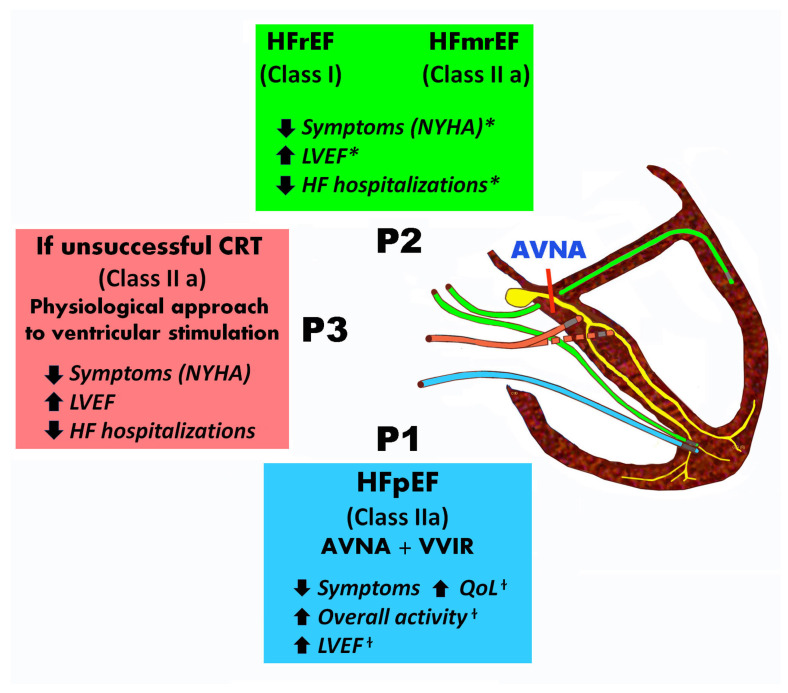
Patients with AF undergoing AV node ablation indications and advantages of different pacing modalities. In patients with symptomatic AF and uncontrolled heart rate, who are eligible for AVJ ablation, CRT is recommended in patients with HFrEF (Class I, Level of Evidence B) and it should be preferred over RV pacing in patients with HFmrEF (Class IIa, Level of Evidence C). In patients with HFpEF there is a Class IIa, Level of Evidence B recommendation for RV pacing. HBP as a more physiological treatment option is indicated in patients who are eligible for CRT, but the coronary sinus lead implantation is unsuccessful (Class IIa, Level of Evidence B). P1:VVIR pacing, P2: CRT pacing, P3:His bundle pacing (HBP), AVNA = AV node ablation, * CRTbeneficial outcomes, ^†^ AV node plus VVIR pacing benefits based on trials, ↓ decrease,↑ increase.

HBPas a treatment option in patients who are eligible for CRT, but where the coronary sinus lead implantation is unsuccessful, is included in the 2021 ESC guidelines on pacing and CRT (Class IIa, Level of Evidence B) [52]. Finally, there is a Class IIb, Level of Evidence C recommendation for HBP with a ventricular backup lead when a “pace and ablate” strategy is indicated for rapidly conducted supraventricular arrhythmia. Figure 1 summarizes the indications and benefits of different pacing modalities combined with AV node ablation.

### 8.3. Left Bundle Branch Pacing (LBBP): A Promising Modality

In recent years, conduction system pacing (CSP) has emerged as it can stimulate the two ventricles simultaneously, providing a more physiological pacing. Except for HBP, LBBP is a new alternativeto CRT which is feasible and safe and has been associated with improved clinical outcomes and echocardiographic parameters [58]. In addition, LBBP is a very challenging and rapidly developing modality with a shorter learning curve compared to HBP according to a review conducted at the Royal Brompton Hospital [59].

A very recent study included patients with HF and refractory AF treated either with BiV (26%) or CSP (HBP 54%, LBBP 20%) plus AV node ablation [60]. Patients in these three groups had similar baseline characteristics. NYHA class improved in both HBP (*p* < 0.001) and LBBP (*p* = 0.008), but not in the BiV group (*p* = 0.096). Similarly, LVEF improved in HBP (39% to 49%, *p* < 0.001) and LBBP (28% to 40%, *p* = 0.041), but it was not increased in the BiV group (*p* = 0.916). In this study, CSP showed significant superiority compared to BiV in terms of clinical and echocardiographic parameters. However, more studies and clinical trials must be conducted in order to obtain more data for patients with AF and HF.

## 9. Conclusions

HF and AF are two cardiovascular conditions affecting many patients around the globe and they are often concomitant. AV node ablation and pacemaker implantation is performed in patients with AF ± HF who do not respond to pulmonary vein isolation or are intolerant to medical treatment.

Compared to medical therapy, the “Pace and ablate” approach shows beneficial effects on clinical outcomes. In addition to a significant improvement in symptoms, especially palpitations and exercise tolerance, this procedure leads to enhanced cardiac performance and EF. Although ablation of the AV junction is shown to reduce mortality in these patients, some early or late adverse effects and complications may occur, including worsening of HF, sudden death and ventricular arrhythmias and permanent AF. However, efficacy and safety of the procedure has been proven in several studies.

Pacemaker implantation is always performed in patients undergoing AV node ablation, as the destruction of physiological conducting system of the heart makes it mandatory. The majority of studies have demonstrated that CRT is the optimal choice of pacing in these patients and is associated with significant improvement in EF and less hemodynamic deterioration, in contrast with classic RV apical pacing.

In recent years, His bundle pacing is an alternative to CRT in case of failure of CRT or in patients who do not respond to it. This approach exploits the patient’s native conducting system in order to cause synchronous ventricular contraction. Recent studies have revealed several positive effects of HBP on patients’ clinical condition and more data will be provided soon by ongoing clinical trials (NCT02805465, NCT02700425). Beyond His pacing, left bundle pacing may confer longer term benefits also, but as of yet no trial data is available.

## Figures and Tables

**Table 1:** Clinical outcomes of AV node ablation [16,17,18,19,20,21,22,23].

First Author	Year of Publication	Design	Number of Patients	Highlights
Wood et al. [16]	2000	Meta-analysis	1181	↑ QOL ↑ Exercise duration ↓ Symptoms ↓ Healthcare use ↑ Cardiac function
Chatarjee et al. [17]	2013	Meta-analysis	7867	↑ QOL ↓ Symptoms ↑ LVEF
Ueng et al. [18]	2001	AVNA + VVIR pacing vs drug therapy	50	↑ QOL ↑ Overall activity ↓ Overall symptoms ↑ LVEF
Brignole et al. [19]	1994	AVNA + pacing vs pacing	23	↓ Symptoms ↑ QOL ↑ LV function
Brignole et al. [20]	1997	AVNA + DDDR pacemaker vs pharmaceutical drugs	43	↓ Palpitations ↓ Effort dyspnea ↓ Exercise intolerance ↓ Easy fatigue
Marshall et al. [21]	1999	AVNA + DDDR/MS pacing vs drug therapy	56	↓ Overall Symptoms ↓ Palpitations ↓ Dyspnea ↑ Psychological general well-being
Brignole et al. [22]	1998	AVNA + VVIR pacing vs drug therapy	66	↓ Palpitations ↓ Exercise intolerance ↓ Effort dyspnea ↓ Chest discomfort ↓ Easy fatigue
Weerasooriya et al. [23]	2003	AVNA + pacing vs medication	99	↓ Peak ventricular rate during exercise and daily activity ↓ Symptoms ↑ QOL

AVNA, Atrioventricular node ablation; QOL, Quality of Life; LVEF, Left Ventricular Ejection Fraction, ↓ decrease, ↑ increase.

**Table 2:** Comparison of BiV and RV pacing.

First Author	Year of Publication	Number of Patients	Design	Highlights
Puggioni et al. [46]	2004	44	LV pacing vs. RV pacing + AVNA	↑ LVEF ↓ MR
Simantirakis et al. [47]	2004	12	LV-based pacing vs. RV pacing + AVNA	↑ LV contractility
Leon et al. [48]	2002	20	BiV pacing vs. RV pacing + AVNA	↑ LVEF ↓ LV diastolic diameter ↓ End-systolic diameter ↓ Number of hospitalizations NYHA class improved
Doshi et al. [49]	2005	184	BiV pacing vs. RV pacing + AVNA	↑ 6-min walk distance ↑ LVEF
Stavrakis et al. [50]	2012	68	CRT vs. RV pacing + AVNA (Meta-analysis)	↓ HF hospitalizations ↑ LVEF

LV, Left Ventricle; RV, Right Ventricle; AVNA, Atrioventricular Node Ablation; BiV, Biventricular; CRT, Cardiac Resynchronization Therapy; LVEF, Left Ventricular Ejection Fraction; MR, Mitral Regurgitation; NYHA, New York Heart Association; HF, Heart Failure. ↓ decrease, ↑ increase.

**Table 3:** ESC recommendations for CRT implantation in patients with HF ± AF.

Class	Level	Recommendation
Class I	A	CRT rather than RV pacing is recommended for patients with HFrEF (EF < 40%) irrespective of NYHA class or QRS width, who are candidates for ventricular pacing for high degree AV block. Patients with AF are also included in this recommendation.
	B	CRT is recommended in patients withHFrEF, symptomatic AFand uncontrolled heart rate who are eligible for AVJ ablation.
Class IIa	B	AVJ ablation should be added in patients with HF and permanent AF who are eligible for CRT, in case of incomplete biventricular pacing (<90–95%) because of conducted AF.
	B	RV pacing should be considered in patients with HFpEF, symptomatic AF and uncontrolled heart rate who are going to undergo AVJ ablation.
	Β	Upgrading from RV pacing to CRT should be considered in patients with conventional pacemaker or ICD who develop HF with LVEF ≤ 35% despite the optimal medical treatment and have a significant proportion of RV pacing.
	C	CRT rather than standard RV pacing should be considered in patients with HFmrEF, symptomatic AF and uncontrolled heart rate who are candidates for AVJ ablation.
	C	CRT should be considered for patients with HF and LVEF ≤ 35% who remain in NYHA class III/IV despite the optimal medical treatment and have AF with inherent QRS ≥ 130 ms.
Class IIb	B	CRT may be considered in patients with HFpEF, symptomatic AF and uncontrolled heart rate who are candidates for AVJ ablation.

## Data Availability

Not applicable.

## Abbreviations

HF-Heart Failure; AF-Atrial Fibrillation; CV-Cardiovascular; LVFP-Left Ventricular Filling Pressure; HFpEF-Heart failure with Preserved Ejection Fraction;HFrEF-Heart failure with Reduced Ejection Fraction; HFmrEF-Heart failure with mildly reduced Ejection Fraction; AV node-Atrioventricular node; QOL-Quality of Life; RR-Relative Risk; SCD-Sudden Cardiac Death; RF-Radiofrequency; LV-Left Ventricle; RRR-Relative Risk Reduction ; CI-Confidence Interval; HR-Heart Rate; LVEF-Left Ventricular Ejection Fraction; VT-Ventricular Tachycardia; VF-Ventricular Fibrillation; ECG-Electrocardiogram; ICD-Implantable Cardioverter Defibrillator; RV-Right Ventricle; BiV-Biventricular; EF-Ejection Fraction;CRT-Cardiac Resynchronization Therapy; ΔLVEFHBP-Difference of LVEF between baseline and HBP; ΔLVEFBiV pacing-Difference of LVEF between baseline and BiV pacing; CSP-Conduction System Pacing; LBBP-Left Bundle Branch Pacing; ESC-European Society of Cardiology.
